# Comparative RNA-seq based transcriptomic analysis of bud dormancy in grape

**DOI:** 10.1186/s12870-016-0960-8

**Published:** 2017-01-19

**Authors:** Muhammad Khalil-Ur-Rehman, Long Sun, Chun-Xia Li, Muhammad Faheem, Wu Wang, Jian-Min Tao

**Affiliations:** 1 Laboratory of Fruit Tree Biotechnology, College of Horticulture, Nanjing Agricultural University, Nanjing, 210095 People’s Republic of China; 2 The State Key Laboratory of Crop Genetics and Germplasm Enhancement, Nanjing Agricultural University, Nanjing, 210095 People’s Republic of China

**Keywords:** RNA-seq, DEGs, Summer buds, Paradormancy, Endodormancy

## Abstract

**Background:**

Bud dormancy is an important biological phenomenon of perennial plants that enables them to survive under harsh environmental circumstances. Grape (*Vitis vinifera*) is one of the most grown fruit crop worldwide; however, underlying mechanisms involved in grape bud dormancy are not yet clear. This work was aimed to explore the underlying molecular mechanism regulating bud dormancy in grape.

**Results:**

We have performed transcriptome and differential transcript expression analyses of “Shine Muscat” grape buds using the Illumina RNA-seq system. Comparisons of transcript expression levels among three stages of dormancy, paradormancy (PD) vs endodormancy (ED), summer buds (SB) vs ED and SB vs PD, resulted in the detection of 8949, 9780 and 3938 differentially expressed transcripts, respectively. Out of approximately 78 million high-quality generated reads, 6096 transcripts were differentially expressed (log2 ratio ≥ 1, FDR ≤ 0.001). Grape reference genome was used for alignment of sequence reads and to measure the expression level of transcripts. Furthermore, findings obtained were then compared using two different databases; Gene Ontology (GO) and Kyoto Encyclopedia of Genes and Genomes (KEGG), to annotate the transcript descriptions and to assign a pathway to each transcript. KEGG analysis revealed that secondary metabolites biosynthesis and plant hormone signaling was found most enriched out of the 127 total pathways. In the comparisons of the PD vs ED and SB vs ED stages of grape buds, the gibberellin (GA) and abscisic acid (ABA) pathways were found to be the most enriched. The ABA and GA pathways were further analyzed to observe the expression pattern of differentially expressed transcripts. Transcripts related to the PP2C family (ABA pathway) were found to be up-regulated in the PD vs ED comparison and down-regulated in the SB vs ED and SB vs PD comparisons. GID1 family transcripts (GA pathway) were up-regulated while DELLA family transcripts were down-regulated during the three dormancy stages. Differentially expressed transcripts (DEGs) related to redox activity were abundant in the GO biological process category. RT-qPCR assay results for 12 selected transcripts validated the data obtained by RNA-seq.

**Conclusion:**

At this stage, taking into account the results obtained so far, it is possible to put forward a hypothesis for the molecular mechanism underlying grape bud dormancy, which may pave the way for ultimate improvements in the grape industry.

**Electronic supplementary material:**

The online version of this article (doi:10.1186/s12870-016-0960-8) contains supplementary material, which is available to authorized users.

## Background

Grape (*Vitis vinifera*) is the most widely grown fruit crop globally. The area under grape cultivation is approximately 7.8 million hectares with a production of about 67.5 million tons. The berries are categorized mainly into table grapes (fresh) and wine grapes (wine), as well as for several value-added products [1]. China is the leading grape-producing country, accounting for 14% of the global grape production [2].

There are several developmental and metabolic processes that occur in the buds and twigs of grape plants during the winter period. These processes include enzyme synthesis, respiration, cell division, photosynthesis, growth stimulator production and growth inhibitor down-regulation. Dormancy is a controlling mechanism that enables woody perennials to adapt seasonal environmental changes and thus affects the following season’s vegetative growth and fruit production. Currently, global warming has a substantial influence on winter chilling accumulation and dormancy release of fruit trees [3]. To ensure sustainable fruit production, it is necessary to investigate the underlying genetic factors responsible for controlling dormancy [4]. Extended dormancy is a key hindrance for the large scale fruit production, including grape, in warm or mild winter regions under temperate and subtropical climates [5, 6]. Several studies have been conducted to determine the association between natural and chemical-induced ED, analyze gene expression during long and short photoperiods, and identify the transcript profile of bud development and signaling of bud dormancy break in grape [7–10]. Dormancy is generally classified into three main types: paradormancy (PD), endodormancy (ED), and ecodormancy (ECD) [11]. PD is the plant growth suspension initiated by factors outside the meristem. It is essentially the effect of one organ on another and involves the dominance of apical buds. ED is regulated by internal growth inhibitors, even under favorable conditions; without exposure to cold temperature for a specific duration (chilling requirement), endodormant buds (EDBs) cannot initiate growth. Exposure to low temperature (2–9 °C) shifts the ED state of the plant to ECD. ECDBs can break and grow when exposed to suitable growth conditions [12]. When EDB’s chilling requirement are fulfilled, the ED is released. EDBs steadily transition to the ECD state, especially under adverse environmental conditions. Summer buds (SB), which are green in color and small in size and grow on one side of winter buds that have no scales, can be observed after dormancy release during the new growth period and remain active for a short time during the transition from dormancy release to early summer dormancy. Like other perennial deciduous fruit plants, grape undergoes a characteristic dormant period during its growth cycle. In southeast China, grape buds fulfill their chilling requirement in the end of February and blossom in following spring. Inadequate cold accumulation hours during this period lead to irregular flowering, which consequently decreases fruit production.

The investigations have been made on dormancy at physiological as well as molecular levels in different deciduous fruits. MADS-box (DAM) genes associated with dormancy-have been isolated to investigate their expression pattern in some fruit plants during dormancy [12, 13]. For example, *DAM1* through *DAM6* have been identified in peach and Japanese apricot [14, 15], while *MADS13-1*, *MADS13-2, MADS13-3*, *PpMADS1* and *PpMADS2* were found in Japanese pear and Chinese white pear (Suli) [16, 17]. The expression profile of these genes during the induction and release of endodormancy indicated that *DAMs* serve as dose-dependent inhibitors of bud break [15]. Additionally, several other genes are involved in the complex molecular network regulating dormancy in deciduous plants. Therefore, segregating single gene is not sufficient for illuminating underlying molecular processed associated with bud dormancy [13].

Recently, the next-generation sequencing (NGS) technology has uplifted the transcriptomic by allowing the RNA-sequencing using cDNA libraries on a large scale. RNA-seq is a highly efficient and modern tool that involves deep sequencing technologies to generate millions of short cDNA reads which is considerably more efficient than microarray analysis [18]. In previous studies, RNA-seq was successfully applied to investigate dormancy based on direct sequencing of cDNAs in several woody plants using 454-pyrosequencing technology [19]. Moreover, in another study the transcriptomic analysis revealed the dormancy-related regulatory pathways involving photoperiod, hormones and circadian clocks [20–22]. Although previous studies have investigated the physiological as well as the molecular mechanism of bud dormancy using the transcriptomic approach in deciduous fruits as well as other crops [13, 16, 23], no attempt has yet been made to study grape bud dormancy at the transcriptomic level.

This study was undertaken to investigate underlying molecular processes regulating bud dormancy in grape and to develop robust foundation for molecular research. RNA-seq technology was used to categorize and characterize the expression profile of differentially expressed genes (DEGs) during three different grape bud dormancy stages. This novel transcriptome and transcript expression profiling data generated through RNA-seq will offer an improved understanding of underlying molecular process of bud dormancy and will pave the way to identifying key genes involved in dormancy for the ultimate improvement of table grape industry.

## Results

### Analysis of RNA-seq libraries

In this study, three cDNA libraries constructed from grape buds during three different stages were sequenced and generated 79.6 million sequence reads. After elimination of low-quality reads and adaptor sequences, 78.5 million clean reads (98.5% of the generated data) were recorded, which were then mapped to the reference genome of grape using HISAT [24]. Furthermore, out of high-quality reads generated from the three samples, uniquely mapped reads were 73.28 to78%, while total mapped reads were 75.16 to 79.33% (Table 1).

**Table 1:** Reads number based on RNA-Seq data in three stages of grape buds

Type	Paradormancy	Endodormancy	Summer buds
Total raw reads	26435288	26770600	26436252
Total mapped reads (%)	20780609 (79.33)	19615041 (75.16)	20449692 (78.02)
Unique mapped reads (%)	20432432 (78.00)	19125122 (73.28)	20125121 (76.78)
Total low quality reads (%)	66206 (0.25)	65536 (0.24)	70588 (0.27)
Multiple mapped reads (%)	348177 (1.33)	489919 (1.88)	324571 (1.24)
Total clean reads (%)	26195652 (99.09)	26097230 (97.48)	26210610 (99.15)

### Differential expression analysis of transcripts

**Fig. 1:**
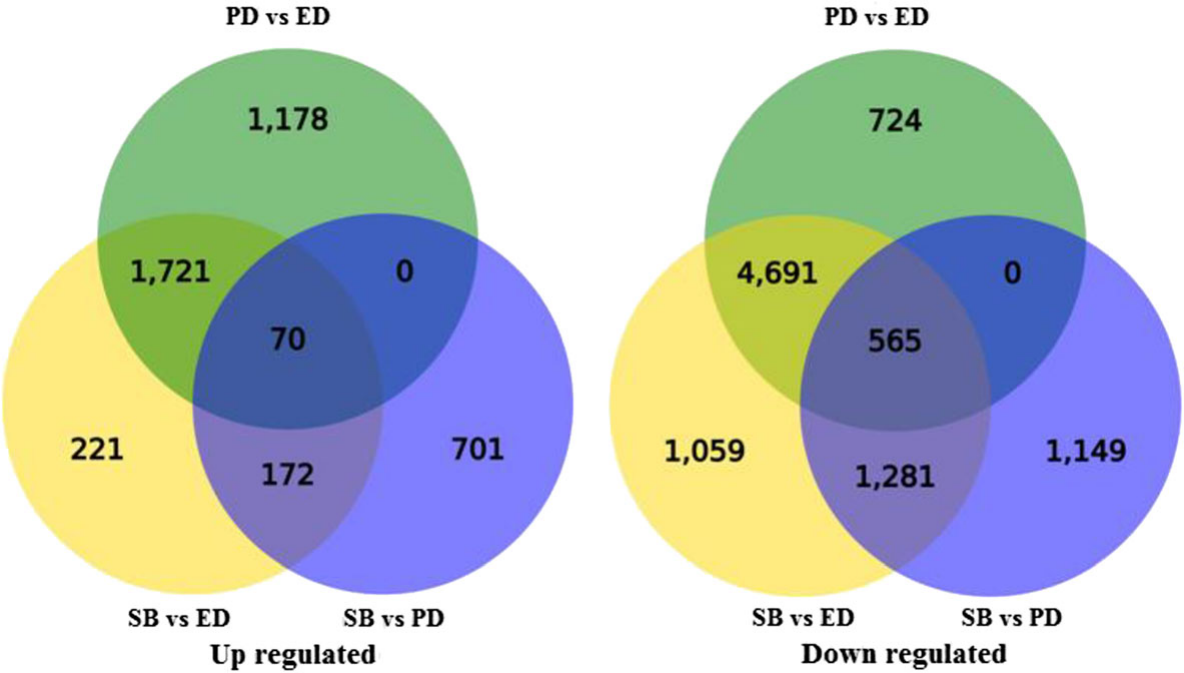
Venn diagram of significantly up-regulated (*left*) and down-regulated transcripts (*right*) in three dormancy stages of grape buds. In this figure, there are 70 up-regulated and 565 down regulated genes were common

To understand and interpret the results of the RNA-seq experiment, the differential expression patterns of transcripts were analyzed among the three different bud dormancy stages. From three different libraries, differential expression analysis identified 943 to 7596 transcripts with significant expression changes (*p* ≤ 0.05 and fold change ≥ 2). The different expression patterns among the three stages revealed that the maximum differences (7596 down-regulated transcripts and 2184 up-regulated transcripts) were examined between the SB and ED stages. In contrast, in the PD vs ED comparison, 2969 transcripts were up-regulated and 5980 were down-regulated, while in the SB vs PD comparison, 943 transcripts were up-regulated and 2995 were down-regulated. Whereas, in comparison between SB and ED stages, the maximum number of 1280 distinctive transcripts was observed, while fraction of unique transcripts were identified in the PD vs ED (1048) and SB vs PD (453) comparisons. Among these, 70 transcripts were commonly up-regulated and 565 transcripts were down-regulated in all three stages of dormancy (Fig. 1, Additional files 1, 2 and 3).

### Cluster analysis of DEGs

**Fig. 2:**
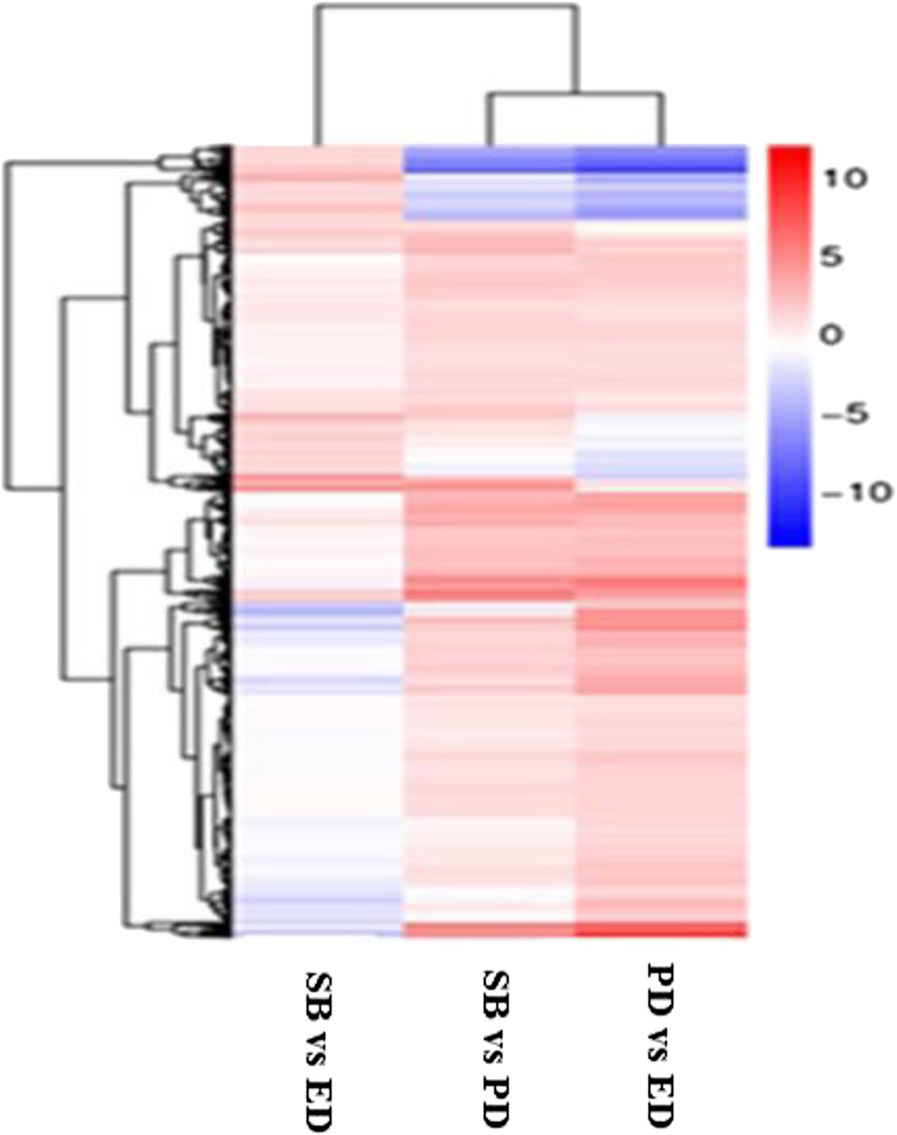
Cluster analysis of gene expression based on log ratio RPKM data. The cluster display expression patterns for a subset of DEGs in three comparisons (PD vs ED, SB vs ED and SB vs PD). Each column represents an experimental condition and each row represents a gene. Red means up-regulated and blue means down-regulated

A cluster analysis of transcript expression patterns with functional enrichment was performed using familiar log ratio values for the transcript expression analysis. The transcripts were arranged into three groups, SB vs PD, SB vs ED and PD vs ED. In the SB vs PD group, 969 transcripts (24.70%) were up-regulated and 2953 transcripts (75.29%) were down-regulated, while in the SB vs ED and PD vs ED groups, 2152 transcripts (54.86%) and 2907 transcripts (74.12%) were up-regulated and 1770 transcripts (45.13%) and 1015 transcripts (25.87%) were down-regulated, respectively. Split plots are shown for each cluster with the data presented as the means of the standard deviation of the RMKM expression values. The cluster analysis grouped up-regulated and down-regulated transcripts separately. A majority of transcripts were up-regulated; while a smaller number of transcripts were down-regulated (Fig. 2 and Additional file 4).

### GO and KEGG analysis of DEGs

**Fig. 3:**
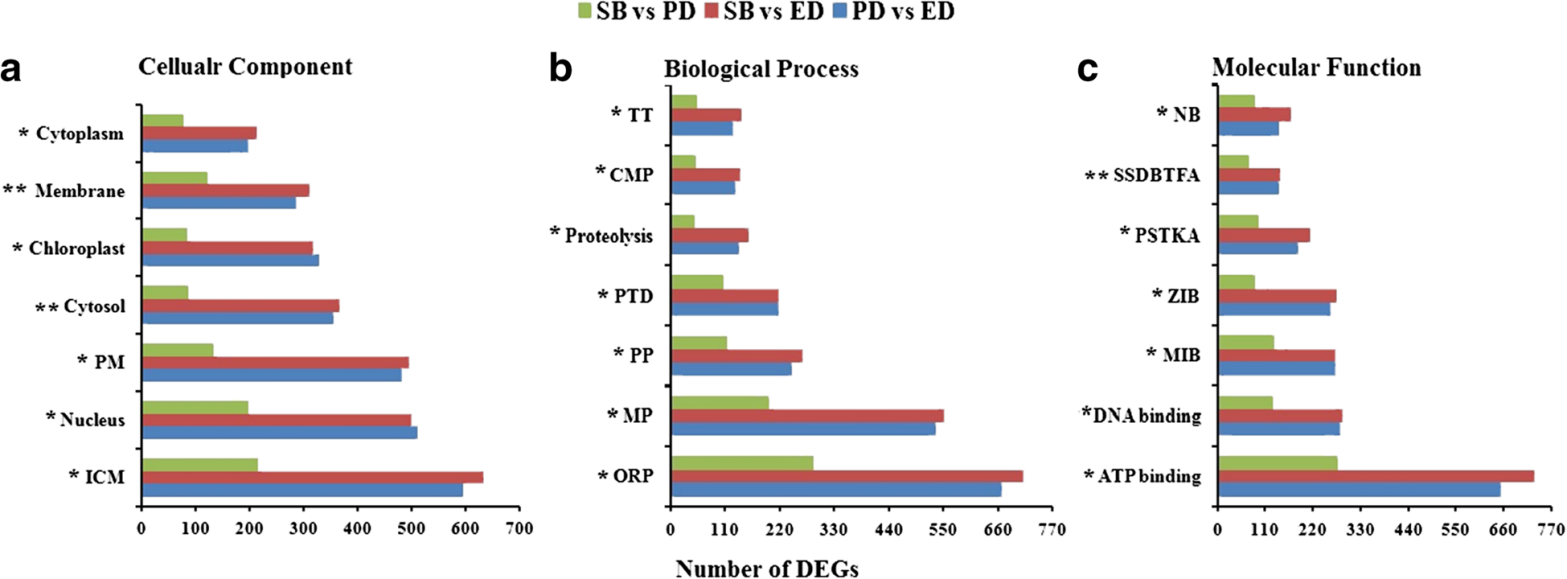
GO distributions of the transcripts differentially expressed among three dormancy stages. GO categories that were significantly enriched, (i.e. *p< 0.05, **p< 0.001) were analyzed with level of significance in pair wise comparison (PD vs ED, SB vs ED and SB vs PD). The transcripts were annotated into three main categories; **a** cellular component, **b** biological process and **c** molecular function. Abbreviations: ICM, Integral component of membrane; PM, Plasma membrane; ORP, Oxidation-reduction process; MP, Metabolic process; PP, Protein phosphorylation; RTD, Regulation of transcription, DNA-templated; CMP, Carbohydrate metabolic process; TT, Transmembrane transport; MIB, Metal ion binding; ZIB, Zinc ion binding; PSTKA, Protein serine/threonine kinase activity; SSDBTFA, Sequence-specific DNA binding transcription factor activity; NB, Nucleotide-binding

**Fig. 4:**
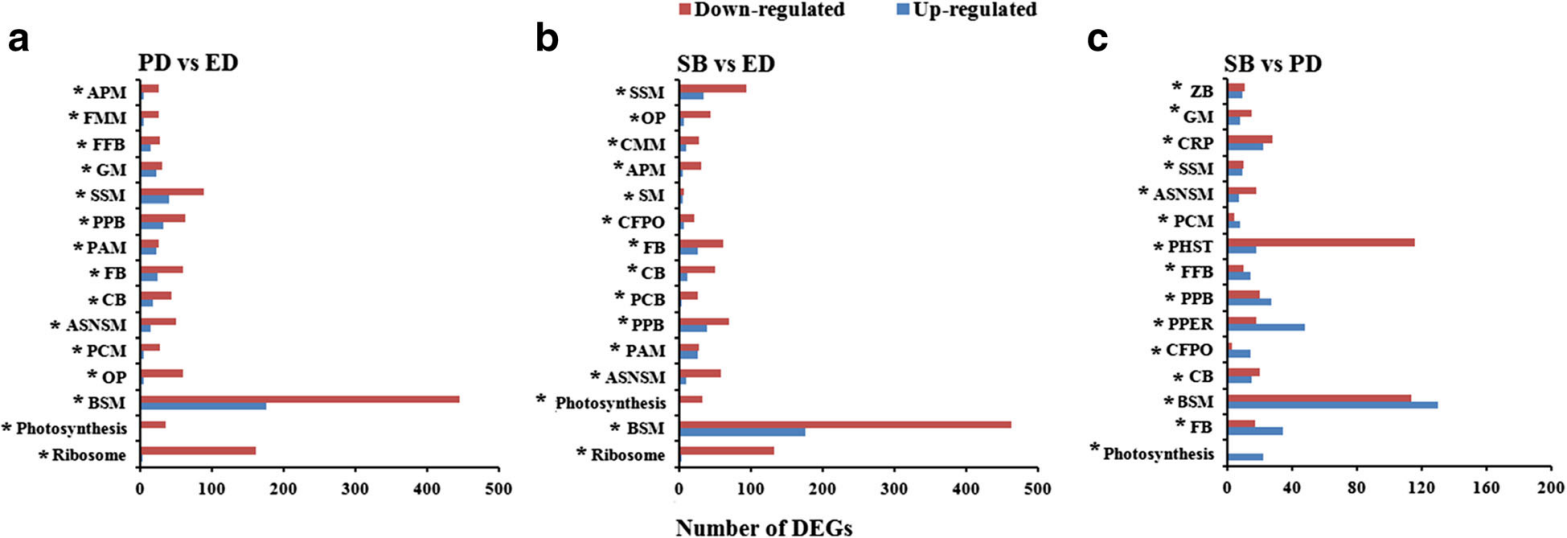
Number of DEGs up and down-regulated in most enriched pathways among three stages of dormancy. Y-axis represents a number of transcripts and X-axis represents enriched pathways. Enriched pathways were significantly enriched (*p< 0.05) during three comparative stages. **a** DEGs number and enriched pathways between PD vs ED. **b** DEGs number and enriched pathways between SB vs ED. **c** DEGs number and enriched pathways between SB vs PD. Abbreviations: BSM, Biosynthesis of secondary metabolites; OP, Oxidative phosphorylation; PCM, Porphyrin and chlorophyll metabolism; ASNSM, Amino sugar and nucleotide sugar metabolism; CB, Carotenoid biosynthesis; FB, Flavonoid biosynthesis; PAM, Phenylalanine metabolism; PPB, Phenylpropanoid biosynthesis; SSM, Starch and sucrose metabolism; GM, Glutathione metabolism; FFB, Flavone and flavonol biosynthesis; FMM, Fructose and mannose metabolism; APM, Arginine and proline metabolism; PCB, Porphyrin and chlorophyll biosynthesis; CFPO, Carbon fixation in photosynthetic organisms; SM, Selenocompound metabolism; CMM, Cysteine and methionine metabolism; PPER, Protein processing in endoplasmic reticulum; PHST, Plant hormone signal transduction; CRP, Circadian rhythm plant; ZB, Zeatin biosynthesis

Gene Ontology based enrichment analysis was carried out using a threshold value (*p*-value ≤ 0.05) to evaluate the major biological functions of DEGs that are further classified into three main categories such as, cellular component (CC), molecular function (MF) and biological process (BP). BP category contained the majority of GO annotations (26,989; 42.15%) followed by MF (21,686; 33.87%) and CC (15,352; 23.97%). The major subcategories along with the analysis of all the transcripts among the three different stages of bud dormancy are shown in Fig. 3. The PD vs ED, SB vs ED and SB vs PD comparisons represent 26,434 (41.28%), 27,559 (43.04%) and 10,034 (15.67%) transcripts, respectively, of the total 64,027 transcripts annotated in GO major categories. A total of 15,352 transcripts were categorized as CC, with 6669 (43.44%) recognized in the PD vs ED comparison, 6642 (43.26%) in the SB vs ED comparison and 2041 (13.29%) in the SB vs PD comparison. Transcripts associated with the CC subcategories integral component of membrane (595; 8.92%, 632; 9.51%, 215; 10.53%) and nucleus (510; 7.64%, 500; 7.52%, 197; 9.65%) were identified in the PD vs ED, SB vs ED and SB vs PD comparisons, respectively. A total of 26,989 transcripts were categorized as BP, with 10,999 (40.75%) identified in the PD vs ED comparison, 11,582 (42.91%) in the SB vs ED comparison and 4408 (16.33%) in the SB vs PD comparison. Transcripts associated with the BP subcategories oxidation-reduction process (667; 6.06%, 712; 6.14%, 288; 2.48%) and metabolic process (534; 4.85%, 551; 4.75%, 199; 4.51%) were recognized in the PD vs ED, SB vs ED and SB vs PD comparisons, respectively. A total of 21,686 transcripts were categorized as MF, with 8766 (40.42%) identified in the PV vs ED comparison, 9335 (43.04%) in the SB vs ED comparison and 3585 (16.53%) in the SB vs PD comparison. Transcripts associated with the MF subcategories ATP binding (653; 7.44%, 730; 7.82%, 277; 7.72%) and DNA binding (282; 3.21%, 290; 3.10%, 128; 3.57%) were recognized in the PD vs ED, SB vs ED and SB vs PD comparisons, respectively (Table 2). A sum of 13,740 DEGs were allocated to 127 pathways (Additional files 5, 6 and 7). Based on KEGG analysis, biosynthesis of secondary metabolites with 1504 transcripts was the most enriched pathway, followed by plant hormone signal transduction (659 transcripts) and ribosome (299 transcripts) in three different dormancy stages (Fig. 4).

**Table 2:** Gene ontology (GO) DEGs number in molecular function, cellular component, and biological process among three dormancy stages

Description	PD vs ED	SB vs ED	SB vs PD	Total
Cellular component	6669	6642	2041	15,352
Bilogical process	10999	11582	4408	26,989
Molecular function	8766	9335	3585	21,686
Total	26,434	27,559	10,034	64,027

### Transcripts related to plant hormone signal transduction and secondary metabolism pathways

In the present study, 1504 transcripts linked secondary metabolism pathways were identified in three dormancy stages. Out of which, 482 and 1022 were up and down-regulated during all three stages of dormancy. 10,312 DEGs were annotated in plant hormone signaling pathways, of which the ABA, gibberellin (GA), and ethylene signaling pathways were further analyzed. Sixteen transcripts were annotated as *protein phosphatase 2C (PP2C)* transcripts, out of which, 11 were up-regulated in the PD vs ED comparison. A large quantity of transcripts abundance of a gene annotated as *serine/threonine-protein kinase (SnRK2)* was lower in the PD vs ED comparison. In GA-responsive pathway, six out of the total 16 transcripts encoding *DELLA* proteins were found to be down-regulated in the PD vs ED comparison, while five transcripts were up-regulated in the SB vs ED comparison. In the ethylene response pathway, two transcripts annotated as *ethylene response receptor (ETR*) were down-regulated in the PD vs ED comparison, while three ETR transcripts were down-regulated in the SB vs PD comparison (Tables 3 and 4). Moreover, differential expression of genes involved in plant hormone signaling pathways was also identified. In the auxin biosynthesis pathway, four out of 15 transcripts encoding *Aux-1* proteins showed up-regulation in the PD vs ED comparison. In the zeatin biosynthesis (cytokinin) pathway, 14 transcripts encoding *CRE1* proteins were identified, with one transcript up-regulated in the PD vs ED comparison and 13 transcripts were down-regulated in the SB vs ED comparison.

**Table 3:** Differentially expressed genes related to plant hormone signal transduction pathway among three dormancy stages

Gene ID	ED (RPKM)	SB (RMKM)	PD (RPKM)	log2	Description
Abscisic acid					
LOC100243241	57.43101	2332.938739	3537.44	−3.38	MLP-like protein 423
LOC100240944	854.1852	454.373409	206.4772	1.36	Probable protein phosphatase 2C 49-like
LOC100853603	2144.361	387.194545	134.615	2.56	Threonine-protein kinase SAPK2-like
LOC100245171	2084.503	1584.199761	792.508	1.5	Serine/threonine-protein kinase SAPK10-like
Gibberellin					
LOC100255710	136.702	6.107169	0.001	4.6	Probable carboxylesterase 8-like
LOC100254982	76.84431	20.764376	11.13357	1.93	Carboxylesterase 1-like
LOC100261706	128.6131	29.314413	15.18215	2.1	Nodulation-signaling pathway 1 protein-like
LOC100253954	2184.805	1520.685199	706.4759	1.1	Scarecrow-like protein 1
LOC100242700	131.8487	68.400298	32.38858	2.05	Scarecrow-like protein 14-like
Auxin					
LOC100246547	464.3014	54.964525	46.55858	2.51	Lysine histidine transporter 1-like
LOC100244496	731.638903	10.51554	720.6459	−3.68	Auxin-responsive protein IAA33-like
LOC100854934	19.4133	131.914861	78.94716	−1.1	An-induced protein 22A-like
Ethylene					
LOC100259653	124.5687	50.07879	18.21858	1.74	Serine/threonine-protein kinase HT1-lik
LOC100257625	1881.472	1408.313281	1212.547	1.09	Serine/threonine-protein kinase HT1-like

**Table 4:** Number of up and down-regulated DEGs related to plant hormone signal transduction pathway

Gene family	Up-regulated	Down-regulated
Lysine histidine transporter 1-like	1	5
AUX/IAA transcription regulator family protein	6	30
TIR 1like auxin family protein	0	13
Histidine kinase binding protein	1	1
CRE1 like family protein	4	28
GIDI family proteins	26	18
DELLA protein SLRI like	15	21
Type 2C protein phosphatases PP2C	17	14
SnRK2 family protein	4	3
Threonine-protein kinase CTR 1 like	4	6

### Validation of DEGs by RT-qPCR

**Fig. 5:**
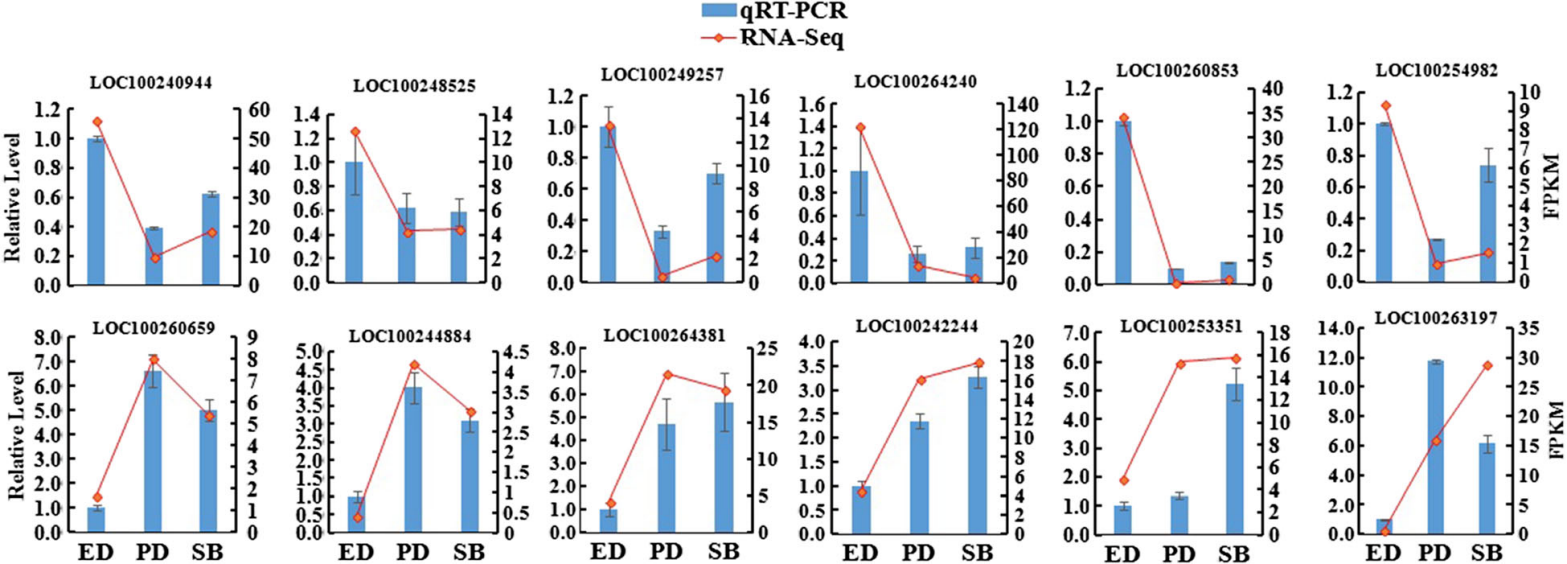
Verification of relative expression levels of DEGs by qRT-PCR. Error bars indicate standard deviation from 3 biological and technical replicates of RT-qPCR. Expression patterns of 12 DEGs related to plant hormone signal transduction pathway by qRT-PCR (blue bar) and RNA-Seq (red line). (1) Gene ID: LOC100240944, Gene Name: *protein phosphatase 2C 49* –like, Gene, Locus ID: VIT_00017639001, (2) Gene ID: LOC100248525, Gene Name: *protein phosphatase 2C 25- like*, Locus ID: VIT_00032793001, (3) Gene ID: LOC100264240, Gene Name: *carboxylesterase 2*, (4) Gene ID: LOC100260853, Gene Name: *carboxylesterase 8*, Locus ID: VIT_00027568001 (5) Gene ID: LOC100249257, Gene Name: *carboxylesterase 120*, Locus ID: VIT_00010672001 (6) Gene ID: LOC100254982, Gene Name : *corboxyleterase1-like*, (7) Gene ID: LOC100260659, Gene Name: *carboxylesterase 12*,Locus ID: VIT_00031776001, (8) Gene ID: LOC100244884, Gene Name: *corboxyleterase 6*, Locus ID: VIT_00025780001, (9) Gene ID: LOC100264381, Gene Name: protein phosphatase 2C 40, Locus ID: VIT_00001129001, (10) Gene ID: LOC100242244, Gene Name: protein phosphatase 2C 15-like, Locus ID: VIT_00011853001, (11) Gene ID: LOC100253351, Gene Name: Protein kinase and PP2C-like, Locus ID: VIT_00025802001, (12) Gene ID: LOC100263197, Gene Name: Protein short root transcript varient X2, Locus ID: VIT_00000107001, (13) Gene ID: LOC100246825, Gene Name: Vv Actin (Reference gene), Locus ID: VIT_00003099001

Twelve DEGs were chosen for RT-qPCR analysis to verify the precision and reproducibility of the transcriptome analysis results. In each case, the qRT-PCR assay results closely related to the transcript levels assessment by the RNA-seq analysis (Fig. 5).

## Discussion

Grape, being one of the most important fruit crops, is globally consumed fresh as well as in the form of several value-added products [1]. Dormancy is a very complex and highly programmed mechanism used by perennial plants to cope with unfavorable environmental conditions. The beginning of dormancy requires sensing and development of regular environmental signals [25]. In grape, a shorter photoperiod and low temperatures cause the alteration of buds into ED [26, 27]. Dormancy can be generally categorized into three dormant states, ED (growth suspension by factors outside the meristem), ED (growth inhibition by internal bud signals) and ECD (growth inhibition by momentary adverse ecological situations) [11]. The molecular and physiological aspects of bud dormancy in grape have been previously examined in several studies [7–10]. This is first ever report on application of RNA-seq technique to classify a large number of transcripts from grape buds of different dormancy stages. Using a transcriptomic approach, we observed that the number and expression profiles of DEGs differed during dormancy stages. A sum of 8949, 9780 and 3983 transcripts were differentially expressed in the PD vs ED, SB vs ED and SB vs PD comparisons, respectively. Transcripts with a like expression patterns might be functionally correlated during bud dormancy. A cluster analysis of DEGs during three comparative dormancy stages was carried out to know the expression pattern of the 11,766 transcripts that were differentially expressed during dormancy stages. The cluster analysis revealed that the most of transcripts were up-regulated while a relatively smaller number of transcripts were down-regulated. Our findings revealed that a number of DEGs were highly expressed in SB vs ED than in the other two stages of dormancy. Previous studies showed that gene activity in black current was minimum at early stages of dormancy and maximum at the moment of bud break [28]. In our study, very high transcript activity in SB vs ED as well as very low activity in SB vs PD was likely due to growth-conducive conditions or signaling from other plants. Additionally, using KEGG analysis, we found that these DEGs belonged to several pathways. Substantial variations were noticed in five pathways, secondary metabolites biosynthesis, ribosome, starch and sucrose metabolism in PD vs ED and SB vs ED stages, while secondary metabolites biosynthesis, signaling of plant hormone and flavonoid biosynthesis pathways were represented in SB vs PD stage of dormancy. Our findings were in consensus with previous work on Chinese pear, in which comparison of transcriptomic analysis between ED and ECD during the whole dormancy cycle showed substantial variations in five KEGG pathways, plant-pathogen interaction, metabolism of ether lipid, ribosome, endocytosis in glycerophospholipid and metabolic pathways [16, 17]. Enriched GO terms recognized in our study, oxidation-reduction process, hormone metabolism and jasmonic acid stimulus, were also in agreement with previous reports [29].

Oxidative stress is proposed to be an important process involved in ED release [30]. Consistent with this perspective, H_2_O_2_ has been reported to be a signaling factor increasing the expression of genes related with release of ED [31]. An increase in H_2_O_2_ levels take place earlier to release ED in grape buds, proposed that H_2_O_2_ could be a signal molecule that triggers gene expression for release of ED. Recent researches have figured out the key role of hydrogen cynamide and calcium signaling in bud break of Perlette grapevines [32]. The higher expression of calcium signaling-related genes corresponds with the optimum bud break potentiation in *V. riparia*, additionally proposing a key role for calcium in the transition from ED to ECD [12]. A significantly down-regulated group of 130 genes was identified during the alteration from ED to ECD at chilling accumulation time in grape and in leafy spurge, and included *proline-rich protein, glutathione S-transferase*, *peroxidase*,, *serine decarboxylase*, *thaumatin, serine carboxy peptidase* and *xyloglucan endo-transglycosylase* [12, 33]. Our data demonstrated the up-regulated expression of *catalase* along with down-regulated expression of some *peroxidase* genes among all three dormancy stages. Down-regulation of *peroxidase* genes and up-regulation of *catalase* genes could enhance or decrease the H_2_O_2_, thus increase release of ED. Therefore, further investigation into the relationship between activity of *catalase* and levels of H_2_O_2_ after ED is required. Generally, metabolic networks are controlled by hormone function and signaling. The involvement of ABA to maintain and promote bud dormancy in woody plants has been projected [34–36]. A gradual decreas of ABA contents during ED to ECD have been reported in leafy spurge and pear buds previously [29, 37] and peaked in poplar after few weeks of short days [38]. Moreover, an ABA related transcript has showed down-regulation during the chilling phase essential for ED release in grape [12]. Similar to these findings, our study showed higher ABA expression in the PD vs ED comparison, while lower expression was observed in the SB vs ED comparison. Based on previous reports, we speculate that ABA might play acrucial role in initiation and maintenance of ED in grape.

Gibberellin (GA) are plant hormones that control several growth processes including seed germination; stem elongation, growth regulation and dormancy. Previous reports have depicted the involvement of GA in bud break, and an increase in GA levels has been considered to be essential for ED release [37]. GA signaling via *GID1* receptors is essential for seed germination in Arabidopsis [39]. Five transcripts in the *GID1* and *DELLA* families were identified and validated by qRT-PCR in the present study. These transcripts also showed different expression patterns during the three dormancy stages. *GID1* family transcripts were up-regulated while *DELLA* family transcripts were down-regulated during the three dormancy stages. Overall, these results suggested that GA was not associated with release of ED activities, with the exception of bud burst initiation.

Basipetally transported auxin is considered as a key signal regulating PD. Cytokinin synthesis is inhibited by auxin. Several genes have been identified in Arabidopsis and pea which involved in auxin-regulated growth inhibition [40]. Cytokinin and auxin signaling have been identified in regulation of PD; however, their involvements in ED are not yet clear [41]. The auxin and cytokinin-responsive transcripts are differentially expressed as plants alteration from PD to ED [29]. In our study, transcripts related to signaling pathways of cytokinin and auxin showed lower expression in all three stages of dormancy. Based on previous studies, we speculate that auxin and cytokinin might be associated in PD and ED regulation of grape buds.

The functional category of identified transcription factors was significantly enriched in the transcript expression profile of this comparative study. Among these identified transcription factors, within the *AP2-like* transcription factor family*, ERF* subfamily with two transcripts was significantly enriched [42], while many of them can regulate the ethylene responses during dormancy and similar responses of *ERF-like* transcription factor have also been reported in poplar [38]. In fact, potato, leafy spurge, and poplar all exhibited the momentary peak in ethylene or ethylene perception that is linked with ED induction as verified by several studies on similar aspect [37, 38, 41]. Another finding on leafy spurge showed contradictory results during PD as revealed by microarray analysis; at least ten ethylene responsive genes were highly induced but were repressed during Ed and ECD [29]. In our study, transcripts related to ethylene signaling pathway showed synchronized expression patterns, with higher *ETR* levels in SB vs PD and lower levels of *CTR1*-like transcripts in PD vs ED. Based on our results, we suggest that ethylene signaling might be involved in endodormancy release.

## Conclusions

As stated above, the results obtained so far allow for the development of a hypothesis regarding the molecular mechanism underlying bud dormancy. By comparing the transcriptomes among three stages, the potential contribution of various pathways in this method became evident. This work implicated several pathways, including plant hormone signaling as well as secondary metabolites biosynthesis. Further confirmation of most enriched pathways and DEGs will be the major emphasis of future studies.

## Methods

### Plant material

Shine Muscat, the most popular table grape cultivar in Japan [43] and China due to its aroma and good taste, was used as the plant material in this study. Four-year-old grape plants were spaced at 6 m × 3 m apart under a rain shelter covered with polyvinyl film and supplemented with drip irrigation at Nanjing Agricultural University Vineyard located in Tangshan Valley, Nanjing, Jiangsu province, China. During the sampling period, plants were not pruned or chemically treated. Buds were harvested on February 02, (ED stage), May 19 (SB) and August 08 (PD stage) in 2015.

The dormancy stages of grape buds prior to constructing gene expression profile were defined as ED, SB, and PD. The growth in the ED stage is stopped due to low chilling exposure and factors within the meristem, while in the PD stage, plant growth is suspended due to factors outside the meristem. SB grows on one side of winter buds having no scales. No bud break was noticed on shoots sampled on 2^nd^ February. These buds were considered to be in ED phase and the collected buds were designated endodormant buds (EDB). The bud samples collected on 19^th^ May and 8^th^ August were designated summer buds (SB) and paradormant buds (PDB), respectively. The samples were instantly frozen in liquid nitrogen and then kept at −80 °C until RNA extraction.

### Preparation of RNA-seq libraries

Total RNA was extracted using Foregene RNA isolation kit (Foregene Co.Ltd, China) according to manufacturer’s instructions. RNA quality was checked with a 2200 Bioanalyzer (Agilent Technologies, Inc., Santa Clara, CA, USA). Total RNA extracted from the three samples collected per dormancy stage was pooled into three sample stages. From each sample, to isolate poly (A) mRNA, 10 μg of total RNA was used to prepare Illumina RNA-seq libraries. From three biological replicates for each stage, each library was pooled by mixing equal quantities of RNA. An insert size of 200 bp was used for sequencing of each library using the Illumina HiSeq^TM^ 2000 system following the manufacturer’s protocol.

### Mapping of reads to the reference genome and gene annotation

**Fig. 6:**
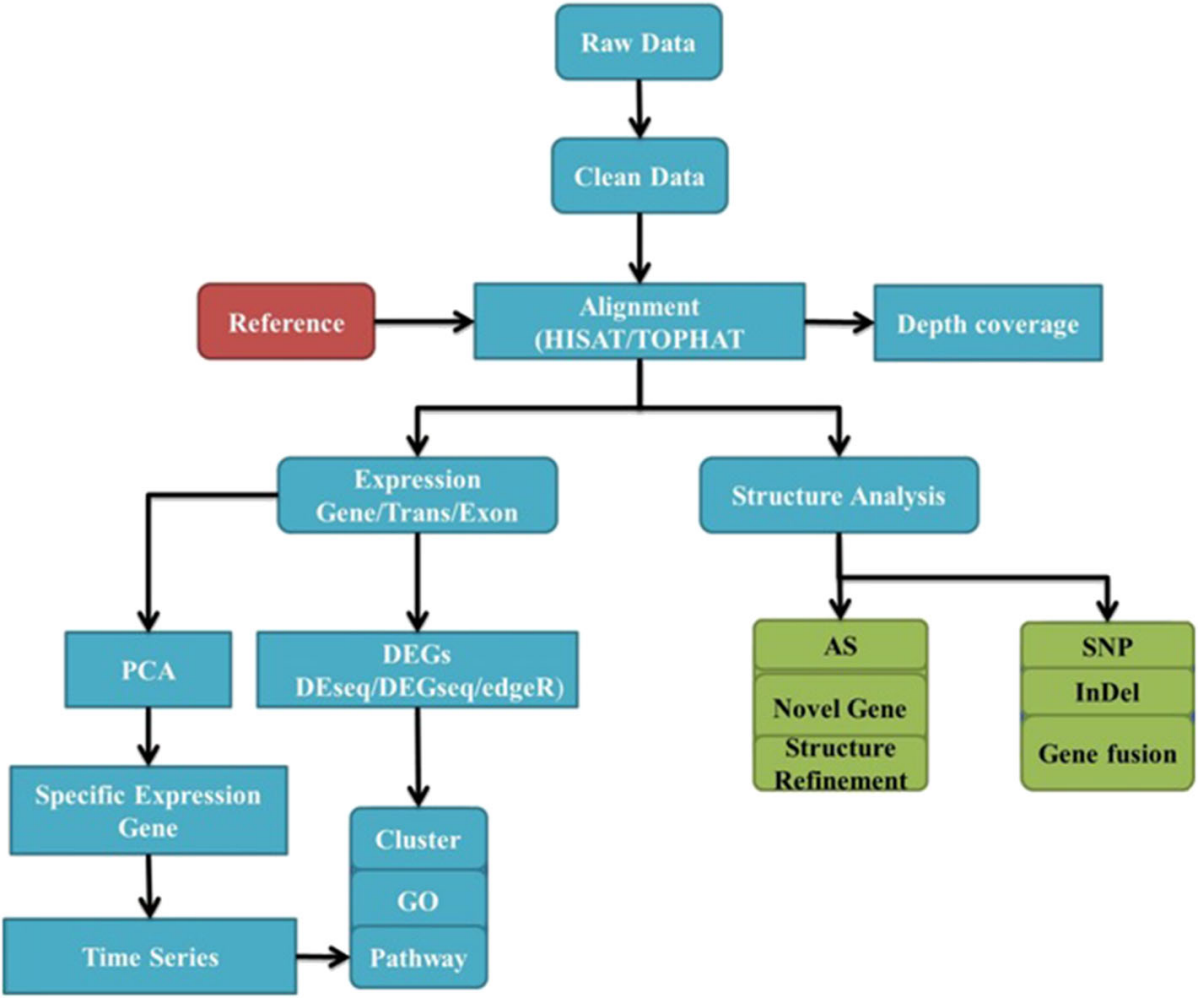
Flow chart of deep sequencing for three sample stages of grape buds

The raw sequence data were filtered by removing adaptor sequences, low quality reads with more than 10% anonymous nucleotides (N) and 50% bases of quality value ≤5 by using hierarchical indexing for spliced alignment of transcripts (HISAT) [24] and standard parameters used for mapping (−−phred64 --n-ceil -q “L, 0, 0.05” -I 100 -X 1000 -t -p 6 --no-una) prior to mapping against a reference grape genome database. Clean reads were mapped to the *Vitis vinifera* reference genome (Assembly accession = GCF_000003745.3; Assembly version = 12X; http://ftp.ncbi.nlm.nih.gov/genomes/Vitis_vinifera/Assembled_chromosomes/seq/ vinifera) using the mapping software HISAT (version 0.1.6). For our data, a read length ≥ 100 bp was used and included reads mapped to the reference genome with ≤ two mismatches [44]. Reads that failed to be mapped were cleaned and mapped to the genome again until a match was found (Fig. 6).

### GO analysis and gene expression evaluation from RNA seq

To compare gene expression levels among three samples, the relative transcript level of each expressed transcript was normalized and calculated to the reads per kilobase of exon model per million mapped reads (RPKM) values [45]. For all RPKM values of each transcript, the cutoff value was determined for shaping gene transcriptional activity based on a 95% confidence threshold. To obtain GO annotations, Blast2GO program was used (version 2.3.5) (https://www.blast2go.com/) for all the transcripts [46]. Further, we performed GO enrichment analysis using GO seq [47] to classify genes or their products into terms (molecular function, biological process and cellular component) that are helpful in understanding the biological functions of the genes.

### Differentially expressed genes (DEGs) and cluster analysis during the three stages of dormancy

DEG seq [48] and DEG seq2 [49] were used to detect the differentially expressed genes. The *p*-value threshold was determined by FDR to account for multiple tests of significance. In this study, FDR threshold ≤ 0.001 and fold change ≥ 2 were adopted to observe the significance of the transcript expression differences [50]. For pathway analysis, all DEGs were mapped to terms in KEGG database and then looked for significantly enriched pathway terms compared to the background genome. KEGG pathways fulfilling the criterion of a Bonferroni [51] corrected *p*-value ≤0.05 were defined as significantly enriched in DEGs. Cluster analyses of gene expression patterns in PD vs ED, SB vs ED and SB vs PD comparisons were performed using R package pheatmap [48]. The sequences obtained from the Illumina sequencing were deposited in the NCBI Sequence Read Archive (accession number, GSE77119).

### Real-time quantitative PCR (RT-qPCR) analysis of DEGs

Twelve genes were selected for validation using quantitative real-time PCR. Primer pairs were designed using Beacon Designer software (Premier Biosoft, version 7.0), which are listed in (Additional file 8). The qPCR reaction was performed in a total volume of 20 μl, containing 1 μl of diluted cDNA, 0.6 μl of reverse and forward primers, 7.4 μl of ddH2O, 0.4 μl of ROX and 10 μl of the PCR master mix (Thermo Fisher Scientific, Waltham, MA, USA). According to the standard protocol of the ABI 7300 system, the amplification program was performed as follows: 30 s at 95 °C, followed by 40 cycles of 5 s at 95 °C for and 30 s at 60 °C. To verify the formation of single peaks and to exclude the possibility of primer dimer and non-specific product formation, a melt curve (15 s at 95 °C, 60 s at 60 °C, and 15 s at 95 °C) was generated by the end of each PCR reaction. All reactions were performed in triplicate, including the non-template control reactions. In addition, the threshold cycles (Ct) of the triplicate reactions for each tested gene were averaged, and then the values were normalized to that of the control V. vinifera Actin gene (accession number XM_010659103) [52].

## Additional files

Additional file 1: Table S1. Differentially expressed genes between paradormancy vs endodormancy. (XLSX 417 kb)
Additional file 2: Table S2. Differentially expressed genes between summer buds vs endodormancy. (XLSX 448 kb)
Additional file 3: Table S3. Differentially expressed genes between summer buds vs paradormancy. (XLSX 187 kb)
Additional file 4: Table S4. Up and down regulated differentially expressed genes in cluster analysis. (XLSX 326 kb)
Additional file 5: Table S5. Differentially expressed genes involved in KEGG pathway between summer buds vs para dormancy. (XLSX 13 kb)
Additional file 6: Table S6. Differentially expressed genes involved in KEGG pathway between summer buds vs endo dormancy. (XLSX 13 kb)
Additional file 7: Table S7. Differentially expressed genes involved in KEGG pathways between paradormancy vs endodormancy. (XLSX 24 kb)
Additional file 8: Table S8. Genes and primer pairs used for quantitative real-time PCR. (DOCX 16 kb)

## Abbreviations

APM: Arginine and proline metabolism
ASNSM: Amino sugar and nucleotide sugar metabolism
BSM: Biosynthesis of secondary metabolites
CB: Carotenoid biosynthesis
CFPO: Carbon fixation in photosynthetic organisms
CMM: Cysteine and methionine metabolism
CMP: Carbohydrate metabolic process
CRP: Circadian rhythm plant
DEGs: Differentially expressed genes
ED: Endodormancy
FB: Flavonoid biosynthesis
FFB: Flavone and flavonol biosynthesis
FMM: Fructose and mannose metabolism
GM: Glutathione metabolism
ICM: Integral component of membrane
MIB: Metal ion binding
MP: Metabolic process
NB: Nucleotide-binding
OP: Oxidative phosphorylation
ORP: Oxidation-reduction process
PAM: Phenylalanine metabolism
PCB: Porphyrin and chlorophyll biosynthesis
PCM: Porphyrin and chlorophyll metabolism
PD: Paradormancy
PHST: Plant hormone signal transduction
PM: Plasma membrane
PP: Protein phosphorylation
PPB: Phenylpropanoid biosynthesis
PPER: Protein processing in endoplasmic reticulum
PSTKA: Protein serine/threonine kinase activity
RNA-seq: RNA sequencing
RTD: Regulation of transcription, DNA-templated
S.B: Summer bud
SM: Selenocompound metabolism
SSDBTFA: Sequence-specific DNA binding transcription factor activity
SSM: Starch and sucrose metabolism
TT: Transmembrane transport
ZB: Zeatin biosynthesis
ZIB: Zinc ion binding
