# Supplementary material for: Comparative RNA-seq based transcriptomic analysis of bud dormancy in grape
Source: BMC Plant Biol. 2017 Jan 19;17:18. doi: 10.1186/s12870-016-0960-8 (PMC5244717; doi:10.1186/s12870-016-0960-8)
Supplement: Additional file 8: Table S8. — Genes and primer pairs used for quantitative real-time PCR. (DOCX 16 kb) [file 12870_2016_960_MOESM8_ESM.docx]

| **Table S8. Differentially expressed genes used for RT-qPCR validation** | | | | | | | | | | | |  |
| --- | --- | --- | --- | --- | --- | --- | --- | --- | --- | --- | --- | --- |
|  |  |  |  |  |  |  |  |  |  |  |  |  |
|  |  |  |  |  |  |  |  |  |  |  |  |  |
|  | **Gene ID** | | **Gene Name** | | **Regulation** |  | **Primer** | | |  |  |  |
|  | LOC100240944 | |  | Protein phosphatase 2C 49 -like | Down |  | **F** | CCGTAGGTGGTTACATTCT | | |  |  |
|  |  | |  |  |  |  | **R** | AATCAGGAACTCATCGTCTT | | |  |  |
|  | LOC100248525 | |  | Protein phosphatase 2C 25- like | Down |  | **F** | AATTCAGAGGAGGACAAGTT | | |  |  |
|  |  | |  |  |  |  | **R** | CCACCACCATTACCGTTA | | |  |  |
|  | LOC100264240 | |  | Corboxyleterase 2-like | Down |  | **F** | GCAAGAGTCCATACCTAAC | | |  |  |
|  |  | |  |  |  |  | **R** | TACCACAGCGTAGAACAA | | |  |  |
|  | LOC100260853 | |  | Corboxyleterase 8-like | Down |  | **F** | GGTTGTGGAGATGATGGA | | |  |  |
|  |  | |  |  |  |  | **R** | GCGTGGAGTCAATGAAAT | | |  |  |
|  | LOC100249257 | |  | Corboxyleterase120-like | Down |  | **F** | GAAGAATGCCTCAGAACAC | | |  |  |
|  |  | |  |  |  |  | **R** | CTGCTTCCACCTCTATCC | | |  |  |
|  | LOC100254982 | |  | Corboxyleterase1-like | Down |  | **F** | GAGTATAGCAATCCGATG | | |  |  |
|  |  | |  |  |  |  | **R** | CTCTATCACATGGTAATCTC | | |  |  |
|  | LOC100260659 | |  | Corboxyleterase1-like | Up |  | **F** | CTTCCTCTCCTCTTCTACATC | | |  |  |
|  |  | |  |  |  |  | **R** | GCAAGCCTATACTCTACTGA | | |  |  |
|  | LOC100244884 | |  | Corboxyleterase 6 | Up |  | **F** | AAGTAGCGTCTCTATGATGT | | |  |  |
|  |  | |  |  |  |  | R | AGTCACAGTTACACCAAGT | | |  |  |
|  | LOC100264381 | |  | Protein phosphatase 2C 40 | Up |  | **F** | CTGAAGAGTTGATGATGATACC | | |  |  |
|  |  | |  |  |  |  | **R** | GAGATGTTGAAGCAGTAGAAG | | |  |  |
|  | LOC100242244 | |  | Protein phosphatase 2C 15-like | Up |  | **F** | AGGAAGACTTAGCATTGTTG | | |  |  |
|  |  | |  |  |  |  | **R** | GGTATTGGAACTATGAACTCTC | | |  |  |
|  | LOC100253351 | |  | Protein kinase and PP2C-like domain containing protein | Up |  | **F** | GAGGAAGGAGATACATACTGAA | | |  |  |
|  |  | |  |  |  |  | **R** | CAAGAACTGGTCGCAATC | | |  |  |
|  | LOC100263197 | |  | Protein short root transcript varient X2 | Up |  | **F** | TCAATCCTACAACTCCAGTA | | |  |  |
|  |  |  |  |  |  |  | **R** | GGAAGAAGAAGAAGAGAAGTC | | |  |  |
|  | LOC100246825 | |  | Vv Actin (Reference gene) |  |  | **F**  TACAATTCCATCATGAAGTGTGATG | | | |  |  |
|  |  | |  |  |  |  | **R**  T TAGAAGCACTTCCTGTGAACAATG | | | |  |  |
|  |  | |  |  |  |  |  | | | |  |  |
